# Learning accurate personalized survival models for predicting hospital discharge and mortality of COVID-19 patients

**DOI:** 10.1038/s41598-022-08601-6

**Published:** 2022-03-16

**Authors:** Neeraj Kumar, Shi-ang Qi, Li-Hao Kuan, Weijie Sun, Jianfei Zhang, Russell Greiner

**Affiliations:** 1 Department of Computing Science, University of Alberta, Edmonton, AB Canada; 2 Alberta Machine Intelligence Institute (Amii), Edmonton, AB Canada

**Keywords:** Machine learning, Statistical methods

## Abstract

Since it emerged in December of 2019, COVID-19 has placed a huge burden on medical care in countries throughout the world, as it led to a huge number of hospitalizations and mortalities. Many medical centers were overloaded, as their intensive care units and auxiliary protection resources proved insufficient, which made the effective allocation of medical resources an urgent matter. This study describes learned survival prediction models that could help medical professionals make effective decisions regarding patient triage and resource allocation. We created multiple data subsets from a publicly available COVID-19 epidemiological dataset to evaluate the effectiveness of various combinations of covariates—age, sex, geographic location, and chronic disease status—in learning survival models (here, “Individual Survival Distributions”; ISDs) for hospital discharge and also for death events. We then supplemented our datasets with demographic and economic information to obtain potentially more accurate survival models. Our extensive experiments compared several ISD models, using various measures. These results show that the “gradient boosting Cox machine” algorithm outperformed the competing techniques, in terms of these performance evaluation metrics, for predicting both an individual’s likelihood of hospital discharge and COVID-19 mortality. Our curated datasets and code base are available at our Github repository for reproducing the results reported in this paper and for supporting future research.

## Introduction

COVID-19, the disease caused by the SARS-CoV-2 virus, broke out in Hubei Province, China, in late 2019, then spread throughout the world^1^. As of 4 May 2021, 219 countries around the world have reported over 151 million confirmed cases of COVID-19, with 3.19 million deaths^2^. Moreover, a large cohort study of over 44,000 patients showed that 19% of the individuals infected by COVID-19 developed severe or critical symptoms^3^, requiring hospitalization. This increased hospitalization rate pushes resource-constrained medical facilities to find effective strategies to prioritize patients for hospital admissions.

Patient triage depends on many factors, including a patient’s age and sex. Although the number of confirmed COVID-19 cases in men is similar to the number of affected women^4,5^, the Global Health 5050 website (https://globalhealth5050.org/, accessed 3 May 2021) shows that male COVID-19 patients have a higher hospitalization rate (11:10), ICU admission rate (18:10), and mortality rate (15:10) than female patients. Additionally, COVID-19 mortality increases with age^6^. For example, in England, for patients under 64 years of age, the infection fatality ratio (IFR) is close to zero, while the IFR increases to 3.1% for patients aged 65–74 and then to 11.6% for patients aged 75 and older^7^. Multiple cohort studies reported similar trends, although the exact IFR values vary across countries^8–11^.

This has motivated many researchers to seek models that can predict, for each individual COVID-19 patient, (1) how long until that hospitalized COVID-19 patient is discharged, and also (2) how long that person will live after contracting COVID-19. Here, we consider ways to learn such models from the current data describing patients and the “outcome”—when each left the hospital (resp., died). This sounds like a standard regression task: learn a model that predicts a positive value (time until the event; perhaps death), given a description of an instance (here, a patient). Unfortunately, many instances in the training data are “censored”—e.g., if a patient was alive after 14 days, but then left the study, we would only know that this patient survived for at least 14 days, but do not know anything more than that. Moreover, “survival datasets” include many such censored patients—indeed, all of our datasets included over 90% censored cases! The field of “Survival Prediction”^12^ explores ways to produce models that can make useful predictions from such datasets—of the form $D = \{[ \mathbf{x}_i, t_i, \delta _i]\}_{i=1\dots M}$ where $\mathbf{x}_i$ is the covariate vector describing the *i*-th patient, $t_i$ is the time of event or censoring, and *i* is the “censoring status” for this patient, which here is 1 if the patient is uncensored (i.e., died at time $t_i$), and 0 otherwise (i.e., was alive at time $t_i$—i.e., will die after time $t_i$). To simplify our notation, we will often just mention “death” as the event throughout, unless we specify “hospital discharge” as the event.

Each of our tasks require estimating the time until an event, for an individual. While there are many types of survival models, they provide different types of information about the patient. For example, one type provides a relative risk score (e.g., Cox-Proportional Hazards model^13^), which is a single number for each patient that is used to estimate which of a pair of people will die first, but does not help us estimate how long either person will live. Other types provide the probability that a patient will live until (at least) a specified time—e.g., the Gail model^14^ provides an estimate of the 5-year risk of developing breast cancer for an individual. However, this single probability value does not provide the personalized breast cancer onset probabilities at any other future time points. The Kaplan–Meier estimator^15^ provides the survival probabilities at all time points, but for an entire population instead of an individual. Unfortunately, as explained below in “Why should we use individual survival distributions?” section, none of these are sufficient for our triage task.

**Figure 1:**
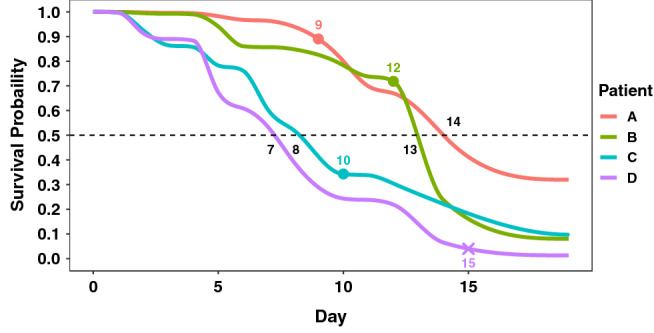
Individual survival distributions of mortality of four patients, computed by the MTLR. We use a solid “$\bullet$” to show when each patient actually died (for patients *A*, *B*, *C*), and an “ $\times$” for the time when a patient was censored (here *D*).

This motivated us to consider Individual Survival Distributions (ISDs)—probabilistic estimates of survival at all future time points for an individual^16^. Figure 1 shows four such ISDs: for each time *t*, $\hat{S}(\,t\, |\, \mathbf{x} \, )$, estimates the probability that patient $\mathbf{x}\, \in \, \{A,B,C,D\}$ will live at least *t* days. Focusing on the teal-colored curve, for patient *C*, we can read off that the prediction that *C*’s median survival time (where that curve crosses 0.5) is 8 days; similarly, there is a 25% chance that *B* (the green curve) will live at least 14 days, and a 75% chance that *A* (the orange curve) will live at least 11 days, etc. From such ISDs, we could easily use the patient ’s specific median survival time for our patient triage tasks. In this paper, we demonstrate the effectiveness of using such ISD models to help perform several important tasks—e.g., prioritizing COVID-19 patients for hospital admission, deciding about future resource needs, etc.—see “Why should we use individual survival distributions?” section.

Our major contributions are (1) curating “survival” datasets describing relevant characteristics of thousands of patients along with their respective time-until-hospital-discharge and time-until-death from COVID-19, obtained from the public COVID-19 epidemiological dataset^17^ (“Data description and preparation” section); and (2) learning survival prediction models from these datasets that can compute ISDs (“Survival prediction algorithms” section), which we demonstrate are useful (“Experimental results” section). We evaluated the effect of (combinations of) multiple covariates (age, gender, location, and chronic disease information) on individual discharge (resp., death) probabilities using the curated datasets. When possible, we also explored whether including additional regional information (demographic and economic) could improve the performance of personalized survival prediction models. Additionally, we evaluated the survival prediction models using several metrics, including the standard C-index, as well as novel metrics—D-Calibration and L1-Margin loss (which assesses individual prediction accuracy). Finally, we are releasing the curated datasets and the code for our survival prediction algorithms for reproducibility and to support the future research in learning personalized survival models for COVID-19 patients.

## Methods

### Data description and preparation

We obtained the datasets used in this paper from the publicly available COVID-19 epidemiological data released by Xu et al.^17^. This dataset was updated regularly until 16 June 2020, and contained epidemiological records of 2.6 million COVID-19 patients, reported between 6 Jan to 16 June 2020, based on the national health reports and other records released by the local and the national health authorities of various countries. For each patient, it included several characteristics including age, sex, symptom onset date, COVID-19 confirmation date, chronic disease (binary variable), chronic disease type, geographic location, and travel history, and where relevant: hospital admission date, specific event date (either death, hospital discharge or last follow-up), and death or discharge status. We excluded all variables that were missing over 80% of the values—here travel history, chronic disease type, etc. We then created three feature sets to obtain our datasets—D1, D2, and D3 (and their end-point-based variants)—from that original dataset, which we then use to train and evaluate multiple individual survival prediction models, each of which can compute the probability (over time) of hospital discharge (resp., death) for each COVID-19 patient included in dataset. Below we provide details of the patient inclusion criteria for each of our datasets. We are also publicly releasing our curated datasets for benchmark comparisons and to facilitate future research in this direction.

#### Dataset D1: Nemanti-like

To compare our approach to Nemati et al.^18^, we curated dataset D1 by using their pre-processing steps. We used the frozen version of the data while Nemati et al.^18^ downloaded the data while it was being updated. This led to differences between their number of patients, versus the size of our dataset D1, even though we used their pre-processing steps. First, we removed any instance missing either age or sex information, and also every patient who died. For the remaining hospitalized instances, we retained only seven variables: age, sex, symptom onset date, hospital admission date, COVID-19 confirmation date, patient status (hospital discharge or censored) and date of hospital discharge. Second, we removed every instance that was missing information for all three dates—symptom onset date, hospital admission date, and confirmation date—while keeping all patients with at least one of these dates available.

Every survival prediction dataset involves three survival variables: start time, time-to-event, and censoring time. Here, we adopted Nemati et al.’s^18^ definitions, given in Table 1 (column 2), to create these variables in D1 that we used to define the real-valued Time variable and binary Censor variable, whose value is 0 if the instance is censored (lost to follow-up and not discharged) or uncensored (discharged). Lastly, we excluded all instances with zero or negative censoring times as well as patients with non-integer age values (e.g., “30–39”, “50–59”). After applying the aforementioned filtering criteria, D1 contained 75,063 patients. For a fair comparison with the results in Nemati et al.^18^, we used only age and sex as covariates, with hospital discharge as the event of interest. Table 1 (column 2) describes D1: time-to-event and censoring time required for survival prediction algorithms, and patient characteristics

**Table 1:** Definition of time-to-event and censoring time for and patient characteristics included in Datasets D1, D2 and D3.

	Dataset D1	Dataset D2 (N = 1718)		Dataset D3 (N = 1422)	
	(N = 75,063)	D2[h]	D2[d]	D3[h]	D3[d]
Event of interest	Hospital discharge	Hospital discharge	Death	Hospital discharge	Death
Start time	First available date	Hospital admission date			
Time-to-event (uncensored cases)	Date of discharge – Start time	Date of discharge – Start time	Date of death – Start time	Date of discharge – Start time	Date of death – Start time
Censoring time (censored cases)	Last available date—start time				
# Uncensored	2127	141	53	135	52
Uncensored (%)	2.80%	8.20%	3.00%	9.40%	3.60%
Mean age (std)	42.27 (17.89)	46.05 (16.95)		46.75 (16.27)	
Sex (male %)	54.34%	55.12%		55.91%	
Patients with chronic disease (%)	Covariate Not included	2.44%		2.88%	

For each patient in D2 (resp., D3), we also include longitude and latitude (resp., longitude, latitude, population density and gross domestic product) information as well. Last available date for a patient is the most recent date among his/her symptom onset date, hospital admission date, COVID-19 confirmation date, date of event (hospital discharge or death).

#### Dataset D2: extra features

Another objective in our study is to explore the effectiveness of additional features, beyond age and sex, in predicting the survival probabilities of hospital discharge (resp., death) for COVID-19 patients. We therefore created dataset D2 to incorporate other covariates, which survival prediction learners can then use to learn models to predict the likelihood of hospital discharge (resp., death). We again started with Xu et al.’s data^17^, and again removed all instances with missing information for any of the following variables—age, sex, chronic disease (binary variable), longitude, latitude, symptom onset date, hospital admission date, COVID-19 confirmation date, date of death or hospital discharge, and death or discharge status. We did not include the type of chronic disease as a covariate because: (1) the missing rate for the type of chronic disease was very high (97.4% of the patients did not have this information), and (2) the text description of chronic disease was vague and difficult to encode into the model.

We used the hospital admission date as the starting time for all patients for each endpoint , leaded to two datasets: hospital discharge D2[h] and death D2[d]. We defined time-to-event as the difference between the date when an event was observed for a patient minus the hospital admission date for that patient. Otherwise (if the patient did not have a hospital discharge nor death), we calculated the censoring time as the period between the last contact with the patient and the hospital admission date. Note we consider dead (resp., discharged) patients as censored when our event of interest is hospital discharge (resp., death) in our analysis. We again excluded all instances with zero or negative censoring times as well as patients with non-integer age values. Finally, we excluded the Argentina patients from D2 because all of these patients (5302) were censored. After applying the aforementioned filtering criteria, the remaining datasets—both D2[h] and D2[d]—each contained 1718 patients. Table 1 (columns 3 and 4) describes the time-to-event and censoring time, for both endpoints—patient death and hospital discharge—required for survival prediction algorithms, as well as patient characteristics.

As we know there are “COVID hotspots” in some geographic regions, we included the location of each patient in D2. However, using the obvious (longitude, latitude) might be problematic during feature scaling/normalization. This motivated us to consider, instead, using transformed latitude and longitude information. Specifically, we applied the following transformation to map the latitude and longitude variables to Cartesian coordinates (*x*, *y*, *z*), considering the center of the Earth as the origin.

1 $$\begin{aligned} {\left\{ \begin{array}{ll} \ x &{} =\ R\ \times \ \cos (\text {latitude})\ \times \ \cos (\text {longitude}) \\ \ y &{} =\ R\ \times \ \cos (\text {latitude})\ \times \ \sin (\text {longitude}) \\ \ z &{} =\ R\ \times \ \sin (\text {latitude}) \end{array}\right. } \end{aligned}$$

where $R (=6371)$ is the approximate radius of the Earth. We compared the performance of survival prediction algorithms when using raw (versus transformed) location information as covariates.

#### Dataset D3—with city information

We created dataset D3 by retaining only the 1422 Asian patients from dataset D2, because we had more detailed geographic information for only these patients, including the city names. We included all of D2’s covariates and also added variables to represent the demographic and economic information for different locations, including (a) city-level population density and (b) country-level gross domestic product (GDP) per capita as covariates (feature (a) was motivated by the observation that, in general, densely populated cities lead to more COVID-19 transmission, and hence more cases and perhaps more deaths; and (b) was motivated by the observation that richer countries have more resources, which typically leads to better treatments with more hospital discharges and fewer deaths). We again considered both end-points: hospital discharge D3[h] and death D3[d]. Again, see Table 1 (columns 4 and 5) for more information about these two datasets.

### Survival prediction algorithms

The literature has provided several types of survival prediction algorithms, including risk scores (e.g., from Cox-Proportional Hazard models), single-time probability models (e.g., the Gail model, predicting 5 year probability), and Kaplan–Meier survival curves. Their limitations, summarized in “Introduction” section, motivated us to explore the more recently proposed individual survival distributions (ISDs) models^16^. This paper assesses the effectiveness of various ISD models in predicting an individual’s probability of discharge from the hospital (resp., death) due to COVID-19. As these ISD models estimate each patient’s specific survival probabilities for all future time points, they can be used for personalized treatment planning and decision making—see “Discussion” section.

We therefore implemented and compared the following ISD models: random survival forest (RSF)^19^, Kalbfleisch-Prentice extension of the Cox model (Cox-KP)^13,20^, multi-task logistic regression (MTLR)^21^, Kalbfleisch-Prentice extension of the gradient boosting Cox machine (GBCM-KP)^22^, accelerated failure time with log-normal distribution (AFT) model^23^, piecewise constant hazard model (PC-Hazard)^24^, Cox-Time model^25^, and DeepHit^26^. However, the main text only briefly describes our top performing model GBCM-KP; Appendix A describes our other top performers: RSF, MTLR, and Cox-KP. Note that this manuscript does not include descriptions nor results of those other models as their performance in our experiments were consistently poor. For the results of all models, see the Appendix or our github repository: https://github.com/kuan0911/ISDEvaluation-covid.

#### Kalbfleisch-Prentice extension of gradient boosting Cox machine

Boosting is a general method for transforming a collection of base learners into one strong learner. The Gradient Boosting Machine (GBM) is a popular boosting algorithm that incrementally trains a set of base models using the gradient residual of the previous stage^22^—iteratively training the *n+1*-st base model to correct the mistakes made by the previous *n* base models, with the hope that the final ensemble of such base models will outperform the individual models. The Gradient Boosting Cox Machine (GBCM) uses the Cox-Proportional Hazard (Cox-PH) base learner to produce each base model in GBM, and uses the Cox-partial likelihood to calculate the gradient residual at each boosting stage^22^. To compute ISDs that use gradient boosting with Cox-PH as the base learner, we need to estimate the time-varying baseline hazard function; our GBCM implementation estimates this using the Kalbfleisch-Prentice estimator (GBCM-KP)^20,27^. Please note that in our experiments GBCM-KP had smaller L1-Margin loss (see “Performance metrics” section) than the GBCM model with Breslow’s estimator^28^—these results appear in Table C.10 of the Appendix. An interesting future direction to explore is to investigate how GBCM algorithm performs with other baseline hazard functions such as B-spline^29^ and P-spline^30^ estimators on the datasets released with this paper.

### Performance metrics

This section briefly introduces the metrics we used to evaluate the performance of our personalized survival prediction models on three aspects: model discriminability (Concordance Index), calibration (D-calibration), and closeness of the actual to the predicted event times (L1-Margin Loss). Since the event rate is very low in all our datasets—around 9% for hospital discharge and 3% for patient mortality (see Table 1)—we do not report the Brier score as it was shown to be of limited clinical utility for such datasets^31^. The next three subsections quickly summarize these metrics. “Should we always pick the model with a better C-index?” section motivates these metrics, especially in the context of our COVID-19 hospitalization tasks. Appendix B provides additional details.

#### Concordance index (C-index)

Concordance index (C-index) is one of the most popular metrics for measuring the discriminability of a survival model, with larger values indicating superior model performance. It first defines the “comparable” pairs of patients and then assesses a survival model’s performance as the percentage of these comparable pairs that are correctly ranked. For a pair of uncensored patients, it computes their respective predicted median survival times and marks that pair as correct if the model’s prediction about who died first matches with reality. To illustrate, note that the median survival time for patient *C* (from Fig. 1) is 8, which is earlier than patient *B*’s (which is 13); this matches the actual event ordering of these two individuals (*C* died at 10 which is earlier than *B*, at 12), and thus this pair will be marked as correct. However, the predictions for *A* and *C* (at 13 and 8, resp.) does not match the order of their deaths (at 8 and 10, resp.).

While every pair of uncensored patients are “comparable”, only those censored patients whose censoring time is greater than the event time of a uncensored patient are considered comparable to that uncensored patient. Here, we see that *C* and the censored *D* are comparable, as *C*’s death at 10 is earlier than *D*’s censoring time of 14. Note C-index ignores censored patients who fail to form “comparable” pairs, and so ignores many “censored + uncensored” pairs, and all pairs of censored patients. We define marginal C-index (mC-index) to include all censored patients in C-index calculation in Section B.3.1 of the Appendix of this paper. More problematic, note that C-index does not measure how close a model’s estimated survival time is to the actual survival time. Nevertheless, this measure is still useful, especially in clinical decision-making where pairwise patient comparisons are important. For example, consider a hospital that has limited number of beds and whose administration has decided to admit patients based on their predicted order of event occurrence. Here, it should prefer a model with high C-index, as it would correctly predict the order of patient deaths, even if its individual time-to-death predictions are inaccurate. Appendix B.1 describes details of how to calculate C-index.

#### D-calibration

Haider et al.^16^ proposed the D-calibration (distribution calibration) test for determining if a model that produces ISDs is meaningful. D-calibration splits the probability-axis of the survival curve into *K* number of equal-sized intervals (e.g., for $K=4$, this would be [0, 0.25), [0.25, 0.5), [0.5, 0.75), [0.75, 1]), and compares the actual number of events with the predicted number of events ($\frac{1}{K}$) within each interval. A model is D-calibrated if the observed number of events within each probability interval are statistically similar to the predicted number of events.

**Figure 2:**
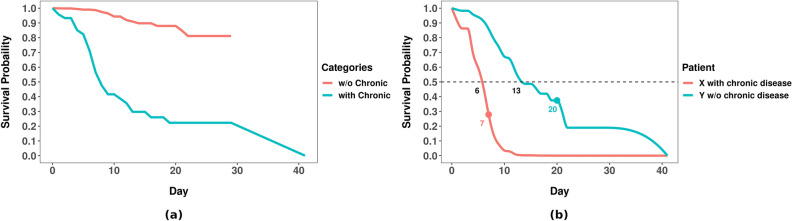
(**a**) KM curves for survival comparison among COVID-19 patients with versus without chronic disease (log-rank *p*-value $< 0.05$); (**b**) GBCM-KP computed individual survival distributions of two example patients from dataset D2[d]. We show the actual time-to-death for each patient with a solid “$\bullet$”; note these are each close to that patient’s predicted median survival time.

As an example, consider the “with Chronic Disease” KM curve in Fig. 2a, and imagine there are 100 COVID-19 patients who qualify—i.e., 100 COVID-19 patients who have some Chronic Disease. As this curve crosses 0.5 at 8.0 days, we expect 50% of these patients to survive at least 8 days—that is what it means to be the median time. Similarly, at least 75% should be alive at 7 days, and 25% at 20 days (i.e., where the curve crosses the 0.75 and 0.25 horizontal lines).

Of course, our methods are dealing with ISDs, where each patient $\mathbf{x}{}_i$ has his/her own survival curve $\hat{S}(\,\cdot \, |\, \mathbf{x}{}_i \, )$, and hence his/her own median survival time $m_i$—i.e., $m_i$ is defined as the time where $\hat{S}(\,m_i\, |\, \mathbf{x}_i \, ) = 0.5$. (e.g., Fig. 1 shows that the median time for *A* (resp., *B*, *C*, *D*) is 14 (resp., 13, 8, 7) days.) Here, we expect 50% of these patients to die before his/her respective median time $m_i$—here we see that *A* and *B* die at 9 and 12 days (which are before their respective median times), and *C* and *D* die after—*C* dies at 10, which is after its median time of 8, and *D* is censored at 15, which means s/he dies after 15 days, which is certainly after the median time of 7. If fact, this figure shows that exactly 1 of these 4 patient died in each quantile—$\hat{S}(\,d_A\, |\, \mathbf{x}_A \, ) \,\in \, [0.75,\,1.0)$, $\hat{S}(\,d_B\, |\, \mathbf{x}_B \, ) \,\in \, [0.50,\,0.75)$, $\hat{S}(\,d_C\, |\, \mathbf{x}_C \, ) \,\in \, [0.25,\,0.50)$, and $\hat{S}(\,d_D\, |\, \mathbf{x}_D \, ) \,\in \, [0.0,\,0.25)$.

D-calibration quantifies this comparison of predicted and actual events within each probability interval. Haider et al.^16^ argued that D-calibrated models produce meaningful ISDs, that can be used for making clinical decisions; see also “Should we always pick the model with a better C-index?” section. Appendix B.2 summarizes how to compute D-calibration.

#### L1-Margin loss

An obvious metric to compare survival prediction models would be “L1 loss”—the absolute difference between the actual and predicted survival times (e.g., median of that patient’s ISD). This requires using the “actual survival time”, which is trivial for uncensored instances, but problematic for censored individuals. Haider et al.^16^ proposed a novel “L1-Margin loss”, which basically “de-censored” the censored patients, by using his/her expected survival time (based on the KM distribution); see Appendix B.3 for more details.

We use this L1-Margin loss to assess how close an individual’s predicted event time (e.g., median survival time) is to his/her actual time of death (or hospital discharge); *n.b.,* this measure is a different metric than the aforementioned calibration and discrimination measures. “Should we always pick the model with a better C-index?” section summarizes why this measure is relevant for our task.

## Experimental results

This section compares the performance of the ISD models, using these three evaluation metrics: C-index, D-Calibration, and L1-Margin Loss. We consider five sets of experiments: one for dataset D1 and two for each of D2 and D3 each of dataset D1, D2[h], D2[d], D3[h] and D3[d]. All evaluations are based on a stratified fivefold cross validation (5CV) procedure, balanced to ensure that event times and censoring rates are consistent across all folds, as suggested in Haider  et al.^16^. Hyper-parameter tuning was done for all models, whenever required, using only the training folds. For C-index and L1-Margin loss, we report the average value across 5CV splits. But for D-calibration, we use test statistics across the 5CV splits to compute a single *p*-value, and consider a survival model to be D-calibrated if the *p*-value is $\ge 0.05$. For clarity, the main text provides only the three “hospital discharge” results , for D1, D2[h] and D3[h]; Appendix C provides the results for the two “death” tasks (D2[d] and D3[d]). The relevant code and our curated datasets are available in the Github repository (https://github.com/kuan0911/ISDEvaluation-covid) to ensure reproducibility and support future research in this direction.

### Experiment 1 (using D1): age and sex as covariates

**Figure 3:**
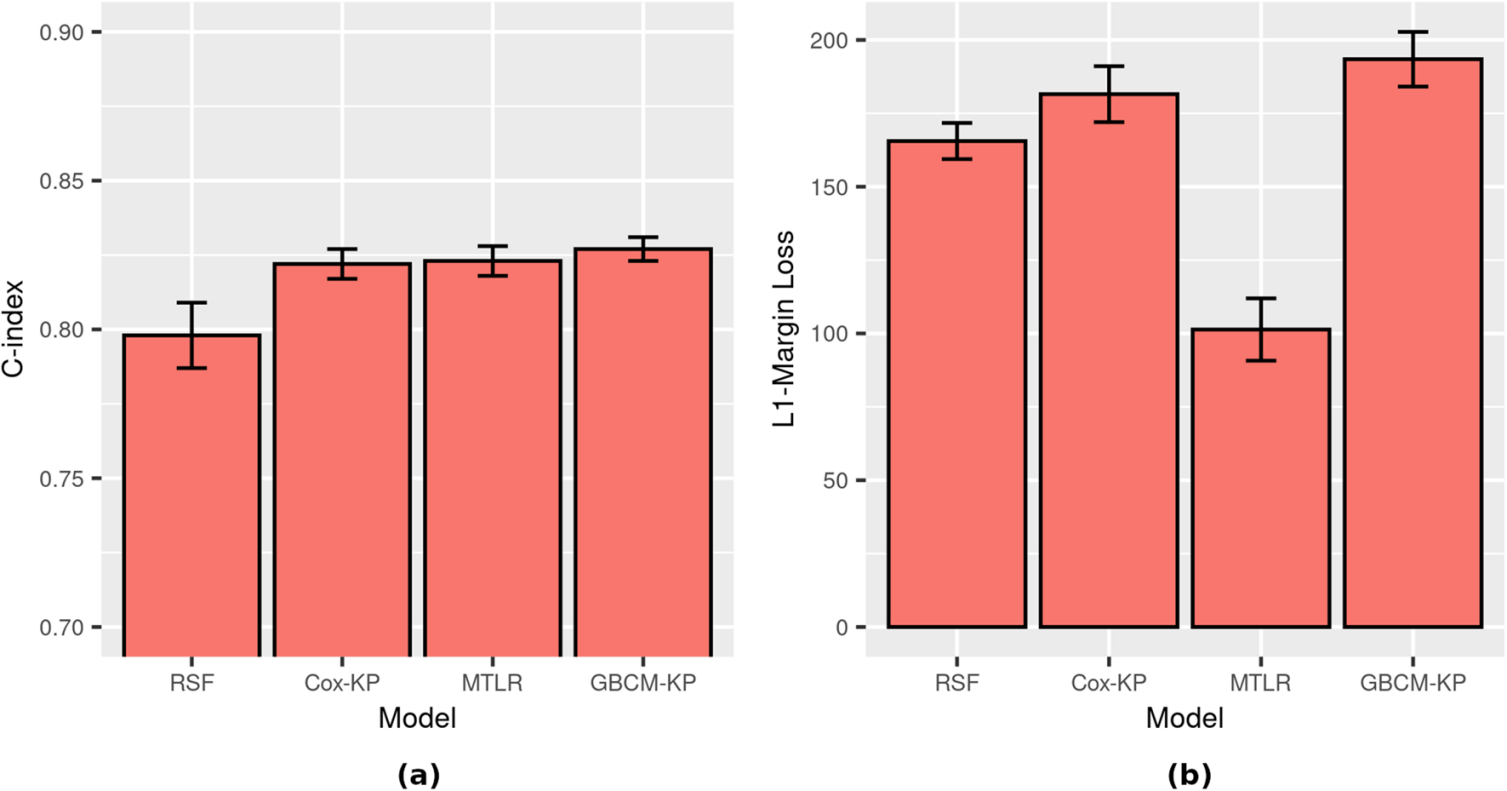
Survival prediction performance on dataset D1 (N = 75,063) for using age and sex as covariates, to predict time until hospital discharge: (**a**) C-index, and (**b**) L1-Margin loss. All four models in this experiment were calibrated (*p*-value $\ge 0.05$). Recall that large values are good for C-index, while small values are good for L1-Margin loss.

This experiment explores the impact of age and sex as covariates on an individual’s likelihood of hospital discharge, using dataset D1 that contains 75,063 hospitalized patients (see Table 1). Figure 3 visualizes the 5CV performance for the models. Table C.1 in Appendix C provides the quantitative results, where the best results among all models are highlighted in bold for each performance metric. We see that GBCM-KP has the highest average C-index and is D-calibrated (*p*-value $\ge 0.05$) but is outperformed by MTLR for L1-Margin loss. Nemati et al.^18^ also reported that GBCM-KP had the best C-index, but they did not compute the other performance metrics.

### Experiment 2: location, chronic disease, age, and sex as covariates (D2[h])

**Figure 4:**
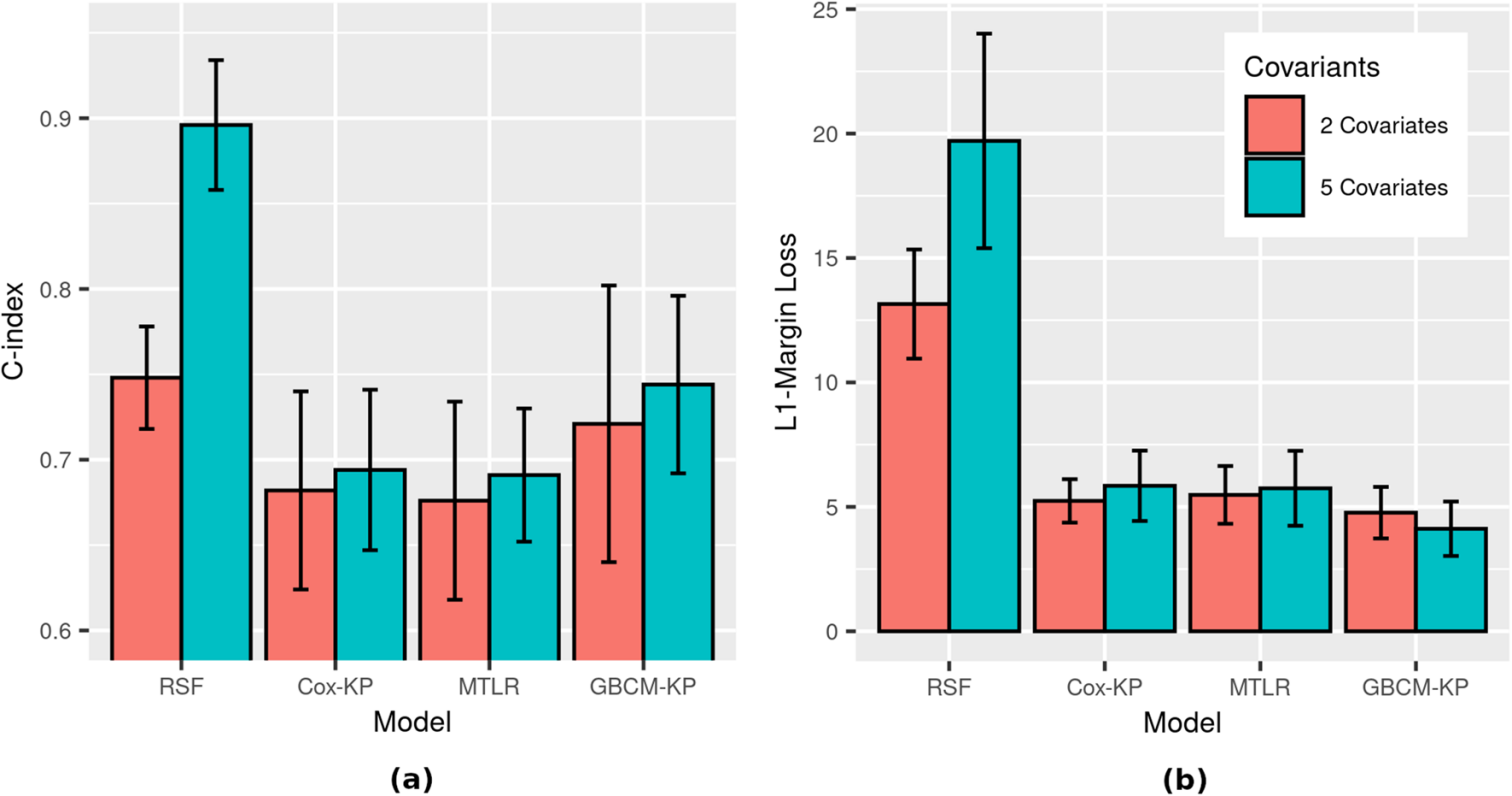
Survival prediction performance on dataset D2[h] (N = 1718) for hospital discharge as event of interest using two covariates (age, sex) versus five covariates (age, sex, chronic disease (binary indicator), longitude, and latitude—(**a**) C-index, and (**b**) L1-Margin loss. The RSF model trained with five covariates in this experiment was not D-calibrated (*p*-value $\approx$ 0) while the other seven models were D-calibrated (*p*-value > 0.05).

Dataset D2 includes 1718 patients who were (1) admitted to hospital, and also (2) included complete information about age, sex, chronic disease (binary indicator), longitude, and latitude, which facilitate more comprehensive analysis of an individual’s likelihood of hospital discharge (D2[h]) and death (D2[d]). Figure 4 shows the 5CV performance for the D2[h] models, where the event of interest is hospital discharge. Table C.2 (resp., Table C.4) in Appendix C shows the quantitative results for various ISD models when the event of interest is hospital discharge (resp., death). Comparing Fig. 4b with Fig. 3b, we see that the L1-Margin loss performance improves considerably when we use additional covariates (chronic disease, latitude, and longitude) on this subset of instances for computing the ISDs—e.g., GBCM-KP going from around 190 days (D1) to around 5 days (D2[h]). To better understand whether the improvement in the L1-Margin is due to the additional features, or to this specific reduced set of instances, we computed all models on the D1 set of features (age and sex), but only on the 1718 D2[h] patients. Here, we found that, again, GBCM-KP performed better than the other models, with 5CV C-index of 0.72 and L1-Margin loss of 4.767—which are respectively $3\%$ and $14\%$ better than their values when all of the features in D2[h] were used for ISD computations in GBCM-KP.

Figure 4 and Table C.2 in the Appendix show that (1) RSF has the best C-index but is not calibrated, and (2) the D-calibrated GBCM-KP model outperforms RSF in terms of L1-Margin loss. So while RSF produced a better ranking for the likelihood of hospital discharge, it failed to produce accurate predictions at the individual level, indicated by the lower L1-Margin loss. These results show that a well-discriminant model can be poorly calibrated^16,32^. This is why, for clinical applications, we might prefer GBCM-KP as the individual survival prediction model since it has the highest discriminative performance (C-index) among all D-calibrated models; note it also had the lowest L1-Margin loss.

We also transformed the longitude and latitude variables into 3 variables, as per Eq. (1), and use this version of the location information instead in the input to the ISD models. We found that there was no significant difference in the performance of the models (Appendix C provides these results for both hospital discharge, D2[h], and death, D2[d].)

### Experiment 3: population density and gross domestic product as additional covariates (D3[h])

**Figure 5:**
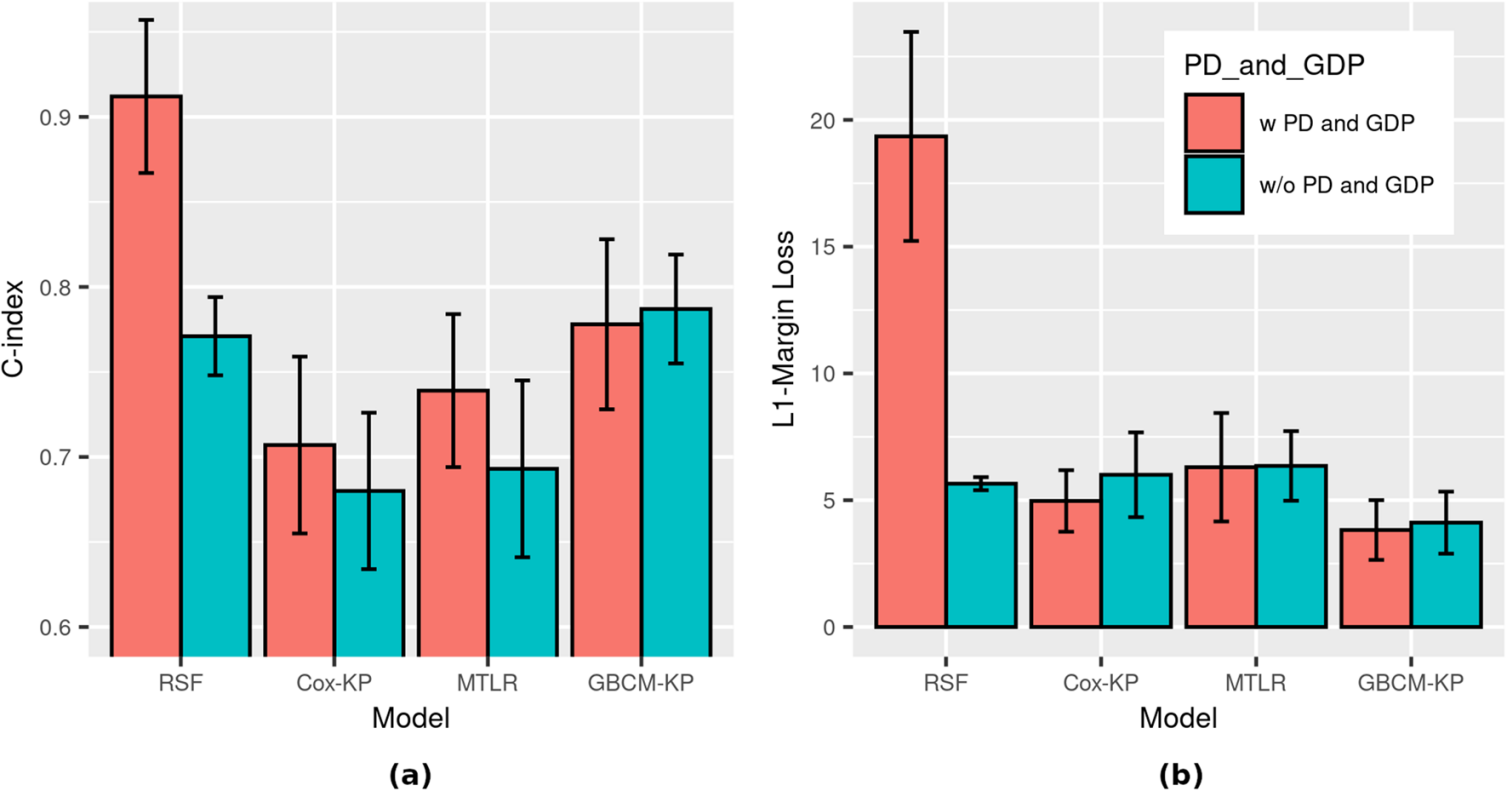
Survival prediction performance on dataset D3[h] (N = 1422) for hospital discharge as event of interest with age, sex, chronic disease (binary indicator), longitude, latitude, and with/without population density (PD) and gross domestic product (GDP) as covariates—(**a**) C-index and (**b**) L1-Margin loss. All eight models in this experiment were D-calibrated (*p*-value $\ge 0.05$).

Our dataset D3[h] contained the subset of the dataset D2[h] patients that included specific city information; these are the 1422 Asian patients. Figure 5 and Table C.3 (resp., Table C.7 in Appendix C) show the 5CV results here for hospital discharge (resp., death) as the events of interest. They also report the results when two different types of feature sets were used to compute ISDs for each patient:

- the first feature set includes all-&-only the variables used in dataset D2[h]: age, gender, longitude, latitude, and chronic disease
- the second feature set includes both the dataset D2[h] variables and also the additional variables: the population density (PD) of the patient’s city, and per capita gross domestic product (GDP) of the patient’s country.

Table C.3 in the Appendix shows that including PD and GDP improves the performance of the survival prediction models (for both C-index and L1-Margin loss). Note that RSF’s C-index performance improves considerably, albeit at the cost of its calibration and accuracy as indicated by D-Calibration and L1-Margin loss values, respectively.

For patient mortality in dataset D2[d] (resp. D3[d]), Appendix C show that the performance of the GBCM-KP models were comparable with RSF models, with only minor differences in C-index, and L1-Margin loss. However, note that all survival models for mortality were D-calibrated and had better performance for other metrics, which may be partly due to very high censoring ($\sim 97\%$) for patient mortality—much higher than the censoring rate for hospital discharge ($\sim 90\%$). Also, we observed no performance improvement when PD and GDP were added as additional covariates, beyond age, sex, chronic disease status, longitude, and latitude in each ISD model for patient mortality in dataset D3[d] (see Appendix C).

## Discussion

### Why should we use individual survival distributions?

Below we describe several situations that benefit from using a model that accurately estimates each patient’s personal survival probabilities at several future time-points. Here, time-invariant relative risk scores (as computed by Cox-PH type models) and single-time survival prediction models (e.g., the Gail model) are not sufficient; instead we need models that predict survival distributions. One such class of models predicts a single survival distribution for *an entire class* (e.g., Kaplan–Meier (KM) models); below we demonstrate the advantages of instead computing *individual* survival distributions (ISDs).

Figure 2a uses KM curves to compare the survival distributions of COVID-19 patients with, versus without, chronic disease, from our D2[d] dataset. We could use this analysis to conclude that this “chronic disease” feature is relevant, as a log-rank test shows that these two survival distributions are statistically different. In fact, these curves show that, *on average*, a COVID-19 patient with a chronic disease will die earlier than the one without. However, we can only make very limited personalized decisions from such KM curves as they only reveal the *average* survival characteristics across a *predefined subgroup*. Another limitation of these KM curves is they cannot distinguish between two “chronic disease” patients, nor between two “not chronic disease” patients, as they are based on only this single feature—i.e., they do not incorporate other covariates, such as the patient’s age, sex, geographic location, etc.

ISDs are a natural extension to those standard KM curves that can involve multiple covariates, which can facilitate personalized healthcare. Figure 2b compares the ISDs, produced by a learned GBCM-KP model, of two COVID-19 patients from dataset D2[d]: X who has a chronic disease and Y who does not. Now, imagine a clinician needs to decide which one of these two patients should be moved to the ICU first? If s/he decides on the basis of the KM curves (Fig. 2a), s/he would move X first, as X belongs to the more-at-risk “chronic disease” group. While this might be appropriate for many pairs of patients, it is wrong in this situation, as in fact, Y died in 7 days, which is before X, who died in 20 days. Figure 2b shows that the GBCM-KP-produced ISDs were more accurate here, as they correctly predicted that patient X will die before patient Y as their predicted median survival times are 6 and 13 days, respectively. Note the GBMC model involved several covariates, which is why we anticipated it would lead to better patient-level decisions.

Of course, we can create many KM curves, each of which provides the survival distribution of the set of patients with certain values for a set of covariates—e.g., one curve for [old, men, w/chronic], another for [old, men, w/o chronic], a third for [old women, w/chronic], ..., and an eighth for [young, female, w/o]. However, this is problematic as it would require a number of KM curves that increases exponentially with the number of covariates; moreover, we would also need an appropriate method for transforming continuous variables (e.g., age) into categorical ones. Alternatively, we can create several high-risk versus low-risk KM curves separately for each covariate. However, these will be difficult to interpret as each individual patient will be modeled with one of the curves associated with each covariate, which might not be consistent—e.g., perhaps a young male patient might be in the low-risk population with respect to age, but in the high-risk group for sex, etc. It is not clear how one would combine these to make a decision about this individual patient, which means that it will pose a problem for personalized clinical decision-making. As our multivariate ISD survival prediction models provide a natural way to personalize these survival distributions, we anticipate they could play a central role in personalized healthcare management and treatment planning in general, and specifically here, for COVID-19 patients.

### Should we always pick the model with a better C-index?

C-index is ubiquitously used for evaluating the performance of survival prediction models. The value of this metric directly reflects the effectiveness of the model in assisting pairwise clinical decisions—e.g., which of the two patients should be admitted to the ICU first. However, it only assesses the correctness of pairwise ordering of patients and does not directly evaluate the survival performance for individual patients and is silent about model calibration and other useful measures—see below.

#### Why do we need L1-margin loss?

**Figure 6:**
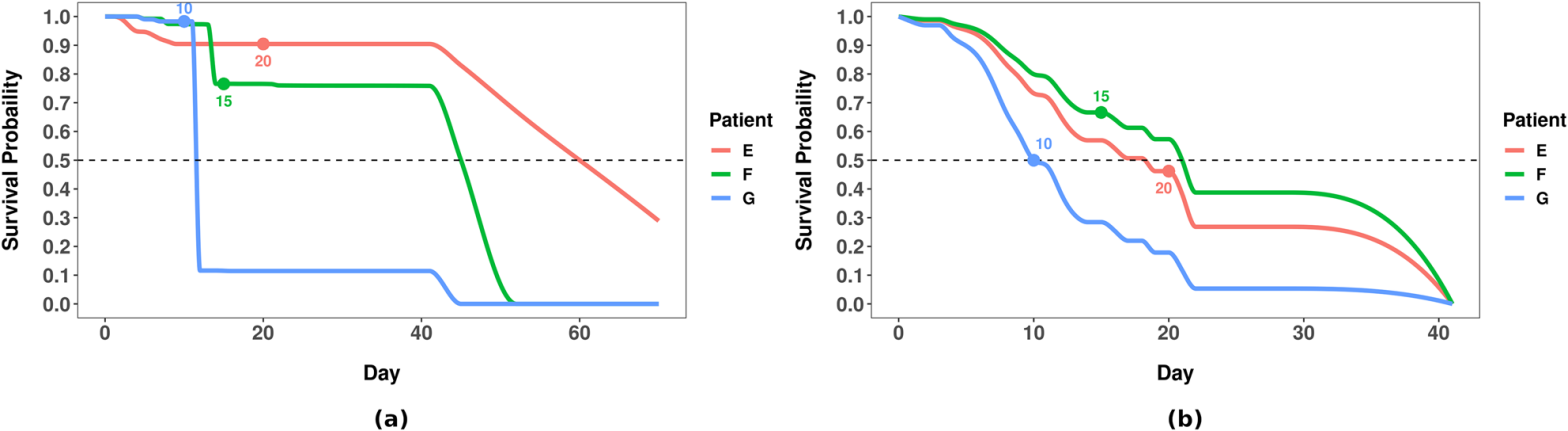
Individual survival curves of the same 3 randomly selected COVID-19 patients from dataset D2[d], for (**a**) RSF, and (**b**) GBCM-KP. Note that the actual death times are shown with solid “$\bullet$” for each patient.

Consider the three hospitalized COVID-19 patients shown in Fig. 6a: patient E died after 20 days in the hospital, patient F after 15 days, and patient G on the 10th day; thus, the correct order of patient death is G followed by F and finally E. The median time obtained from the ISDs computed by our trained RSF model was 60, 45, and 12 days for patient E, F, and G, respectively. As the RSF model correctly predicted that patient G dies before patient F, who in turn dies before patient E, it has a perfect C-index (= 1.0), even though the individual predictions are inaccurate compared to the actual death times. However, we see that RSF has overestimated the median survival time for patients E and F—predicting median survival times of both that is beyond 41 days (which is the maximum survival time in dataset D2[d]). This overestimation is problematic for patient triage tasks if a clinician uses a threshold on the median survival time to prioritize patients for ICU admissions—e.g., sending all patients who are likely to die within the next 30 days to the ICU. If so, a clinician who used RSF would admit only patient G but neither E nor F. Figure 6b shows the ISDs of these patients produced by the GBCM-KP model; clearly GBCM-KP’s predicted median survival time matches the reality more closely than RSF as it has smaller average L1-Margin loss than RSF’s predictions for this example (3.3 versus 24.0). If the clinician’s ICU admission decision is based on these GBCM-KP ISDs, then s/he would admit all three patients, which are the correct decisions. Thus, C-index alone might not lead to the appropriate personalized decisions regarding patient care and management.

#### Why do we need D-calibration?

Another problem with C-index is that it does not measure model calibration, an important criteria for clinical acceptance and reliability of survival prediction models^32^. We used D-calibration, a novel metric proposed by Haider et al.^16^, to assess the calibration of ISD survival models (see “Performance metrics” section and Appendix B.2 for details.) Here we will illustrate, through an example, how D-calibrated survival models could be used for making useful clinical decisions.

Imagine that a hospital with 4 bed has admitted the four COVID-19 patients of Fig. 1: admitting patient *A* on day 1, B on day 3, and both C and D on day 6. Hence, on this 6th day, assuming no one has left (checked out, died, etc.), the hospital is now fully occupied with these four patients. The hospital management is anticipating new patients arriving in the future—say on day 10—and so now needs to decide if they should add more beds for this future. This depends on whether the status of those current patients. We can estimate the expected number of patients in hospital on day 10 as the sum of their respective “still in the hospital” probabilities, i.e., $\hat{S}(\,9\, |\, \mathbf{x}_A \, ) + \hat{S}(\,7\, |\, \mathbf{x}_B \, ) + \hat{S}(\,4\, |\, \mathbf{x}_C \, ) + \hat{S}(\,4\, |\, \mathbf{x}_D \, )$, where each $\mathbf{x}_i$ represents the covariates for patient $i\in \{A, B, C, D\}$, here using the $\hat{S}(\,t\, |\, \mathbf{x} \, )$ ISDs of Fig. 1 (technically, we should use the conditional distributions—e.g., $\hat{S}(\,9\, |\, \mathbf{x}_A, \text{ Discharge } > 4 \, )$—as we know they have not been discharged prior to the time of the prediction—“today”. Fortunately, this is trivial to compute; see [^16^, Theorem B.1]).

Now, the hospital management could use this “expected number of hospitalized patients” and local incidence rate of COVID-19 at a specific future time point to determine the hospital’s bed requirements. The “expected number of hospitalized patients” is meaningful and reliable for clinical decision making only if the underlying survival prediction model was D-calibrated^16,33^. Note that the GBMC model was indeed D-calibrated in our experiments (“Experimental results” section) and hence, its ISDs could be reliably used for hospital resource management.

Notes: (1) In general, we would also need to consider other ways each bed could be freed-up—by death, etc. These too could be estimated using survival techniques (perhaps using competing risk models^26^). (2) KM distributions tend to be D-calibrated as well, but while their estimates would be useful, they are not as personalized, and hence not as accurate, as using a D-calibrated ISD model. (3) The more traditional single-time calibration (called 1-calibration in^16^) is not sufficient here, as we need to consider multiple different time-points to deal with a single “target date”—here, for day 10: 9 days for patient *A*, 7 for *B* and 4 for *C* and *D*. Also, for each target date, we will need to consider another set of 3 future-dates (for the 4 patients): e.g., for day 15, would need to consider 14 days for *A*, 12 for *B* and 11 for *C* and *D*; etc. (see also Appendix B.2 and Haider et al.^16^.)

#### Proposed criteria for model selection

The above examples show that a useful ISD-producing survival prediction model should ...

- be D-calibrated to ensure that the ISDs computed using that model are correct and reliable for trustworthy clinical decision making—e.g., for estimating how many hospital beds will be occupied at several future time points;
- have high discriminatory performance (i.e., high C-index), to ensure correct *comparative* decisions—e.g., which patients are predicted to die relatively earlier than others to prioritize ICU admissions if there are limited number of resources (e.g., ICU beds); and
- have low L1-Margin loss, to make accurate individual decisions—e.g., to determine which individual patients qualify for ICU admission based on a threshold on each patient’s predicted median survival time.

Note that others, including Van Calster et al.^32^, similarly argue that discrimination is not the only criteria—i.e., we should also consider calibration. Although the criteria to identify the appropriate model depends on the specific application, as a general rule, we recommend selecting the survival model that has the highest C-index among the D-calibrated models, and preferring the model with the lowest L1-Margin loss among models with comparable C-index values. For most of our experiments, this criteria identifies the best model as GBCM-KP.

## Conclusion

This paper has demonstrated the effectiveness of survival prediction algorithms that compute individual survival distributions for predicting personalized likelihoods (over time) of hospital discharge (resp., death) for COVID-19 patients. We curated multiple datasets from COVID-19 epidemiological data^18^ to evaluate the effect of various (sub)sets of covariates—including age, sex, chronic disease history, geographic, demographic, and economic information—to estimate the time to hospital discharge, and to death. We evaluated several different individual survival prediction algorithms for both discrimination and calibration through three evaluation metrics: C-index, D-Calibration, and L1-Margin loss. Our results showed that the GBCM-KP learning algorithm produced models that had the highest C-index among all D-calibrated models and also had the lowest L1-Margin loss, for predicting both hospital discharge and patient mortality in most of the experiments. This paper also described the way these individual survival distributions can help in delivering personalized care to individuals infected with COVID-19. Finally, we also demonstrated the importance of calibrated survival models for individual survival prediction and discussed the limitations of models that are discriminatory but poorly calibrated. We also compared the survival prediction models for L1-Margin loss to assess the quality of individual predictions—a measure that is different from both discrimination (C-index) and calibration (D-calibration) measures.

## Supplementary Information

Supplementary Information.

## Data Availability

We have publicly released our curated datasets and codebase for reproducibility of the reported results and for supporting the future research in developing individual survival prediction algorithms for COVID-19 patients (see: https://github.com/kuan0911/ISDEvaluation-covid).
