# Supplementary material for: Learning accurate personalized survival models for predicting hospital discharge and mortality of COVID-19 patients
Source: Sci Rep. 2022 Mar 16;12:4472. doi: 10.1038/s41598-022-08601-6 (PMC8927593; doi:10.1038/s41598-022-08601-6)
Supplement: Supplementary file 1 — Supplementary Information. [file 41598_2022_8601_MOESM1_ESM.pdf]

Appendix: Learning Accurate Personalized Survival Models for Predicting Hospital Discharge and Mortality of COVID-19 Patients

Neeraj Kumar ∗, Shi-ang Qi ∗, Li-Hao Kuan, Weijie Sun, Jianfei Zhang and Russell Greiner (∗ joint first authors)

Note: Reference numbers refer to the reference list in the main text.

Appendix

A Details of ISD Algorithms

A.1 Random Survival Forest

Random forest is a useful bagging-based tool for learning regression/classification models that reduces the variance of many survival trees [31]. The Random Survival Forest (RSF) approach extends random forest by incorporating right-censored survival data into the base estimators [19], by growing a survival Classification and Regression Tree (CART) for each bootstrap sample by randomly picking a small subset of candidate variables for consideration at each node of the tree. A node is split using the candidate variable that maximizes the survival difference between two child nodes based on the long-rank splitting criteria. The tree stops growing when a leaf node has less than a threshold number of unique uncensored instances. Once trained, each test instance is passed through each tree until reaching a leaf node; it then assembles the instances associated with that leaf node. It then runs the Kaplan-Meier (KM) estimator on these the leaf-associated instances, to produce a single survival curve for that test instance, and iterates over each tree. The ensemble KM curve estimator for a test individual is then computed by averaging across all the Kaplan-Meier curves produced by all of the trees.

A.2 Kalbfleisch-Prentice extension of Cox-Proportional-Hazard Model

Cox-Proportional-Hazard (Cox-PH) is a widely used model for predicting an individual's risk using a set of covariates $\mathbf{x}_i = [x_{i1}, \ldots, x_{iJ}]$ [13]. It is a semi-parametric model with the proportional baseline hazard assumption that estimates an individual's hazard as:

$$h_{\text{CoxPH}}(t, x_i) \quad = \quad \lambda_0(t) \ \exp \sum_{j=1}^{J} x_{ij} \beta_j, \tag{1}$$

where β_j is the learned weight for j-th covariate and $\lambda_0(t)$ is the time-dependent baseline hazard function, which is usually unspecified (as it is the same across all patients). The value obtained from the exponential of the Cox-PH model is treated as personalized *time-invariant* risk score. The Kalbfleisch-Prentice extension (Cox-KP) fits a non-parametric baseline function for computing $\lambda_0(t)$, which can then be used to generate the individual survival curve across all time-points [20]. This paper compares these Cox-KP models with other ISD models.

A.3 Multi-task Logistic Regression

Yu *et al.* proposed the multi-task logistic regression (MTLR) model to compute ISDs by overcoming the limitations of the proportional hazard assumption of the Cox-PH model [21]. MTLR divides the study time period into discrete-time intervals and trains essentially a logistic regression model for each interval. The output for each logistic regression estimator is a binary variable $y_i \in \{0, 1\}$, indicating whether this individual is alive in that time interval. The complete ISD is then calculated by combining the logistic regression computation of each interval:

$$f(\ [y_i, y_2, \ldots, y_m] \,|\, \mathbf{x}\ ) \quad = \quad \frac{\exp \sum_{i=1}^{m} y_i (\theta_i \cdot \mathbf{x} + b_i)}{\sum_{j=0}^{m} \exp \sum_{k=j+1}^{m} (\theta_k \cdot \mathbf{x} + b_k)}, \tag{2}$$

where each θ_i represents logistic regression parameters and b_i is the bias term, $\mathbf{x}$ is a vector of an individual's input covariates, and $y = [y_i, y_2, \ldots, y_m] \in \{0, 1\}^m$ is an individual's event indicator for each interval. Note that all the subscripts in this equation represent the index of the time interval.

B Evaluation Metrics

This paper uses the following performance metrics:

B.1 Concordance Index (C-index)

Concordance index, also known as C-index, considers all pairs of “comparable” instances, and computes the proportion of these pairs of instances whose actual pair-wise survival ordering, matches the predicted ordering of the survival model as:

$$\text{C-index}(\ \hat{S}(\cdot\,|\,\cdot),\ D\ ) \quad = \quad \frac{\sum_{i:\delta_i=1}\sum_{j:t_i<t_j}\mathbb{1}[\text{Med}(\hat{S}(\cdot\,|\,\mathbf{x}_i)) < \text{Med}(\hat{S}(\cdot\,|\,\mathbf{x}_j)))]}{\sum_{i:\delta_i=1}\sum_{j:t_i<t_j}1} \tag{3}$$

where the denominator is the number of “comparable” pairs, $\text{Med}(\hat{S}(\cdot\,|\,\mathbf{x}))$ is the median survival time of the survival distribution $\hat{S}(\cdot\,|\,\mathbf{x}))$, $\mathbb{1}[\phi]$ is the indicator function, which is 1 if the proposition ϕ is true (otherwise 0), and $\delta_i = 1$ means the i-th patient is uncensored. A pair of patients is “comparable” if we can determine who died (or was discharged) first – *i.e.*, if both are uncensored, or when one patient is censored after the observed (uncensored) event time of the other; this corresponds to the set of ordered pairs of indices. The C-index score is a real value between 0 to 1, where 1 means all comparable pairs are predicted correctly. C-index only measures the discriminative ability of a survival model and considers only a (possibly small) fraction of all pairs of patients.

B.2 D-Calibration

Standard “1-calibration” measures the deviation between the observed and the predicted probabilities, over all instances, for a single time point – for example, the probability of dying from COVID-19 in 10 days. But this only considers a single time; instead, distribution calibration (D-calibration) is a way to evaluate the calibration of survival prediction models that produce ISDs (which give probabilities for all future times) [16]. Given a dataset $D = \{[\mathbf{x}_i, t_i, \delta_i = 1]\}$ of uncensored patients, a survival model Θ, and any interval $[a,b] \subset [0,1]$, let

$$V_{\Theta,D}([a,b]) = \{[\mathbf{x}_i, t_i, \delta_i = 1] \in D\,|\,\hat{S}_\Theta(t_i|x_i) \in [a,b]\} \tag{4}$$

be the subset of patients in D whose time of death is assigned a probability (by Θ) in the interval $[a,b]$. A model Θ is considered D-Calibrated if this proportion of patients $\frac{V_{\Theta,D}([a,b])}{D}$ matches $b-a$; *e.g.*, $V_{\Theta,D}([0,0.5])$ should include half (*i.e.*, $0.5-0$) of the patients, and $V_{\Theta,D}([0.5,0.75])$ should include $0.75-0.5 = \frac{1}{4}$ of the patients, etc.

We can compare the distributions of predicted and observed proportions of events using χ^2 goodness-of-fit test [32]. A well-calibrated model will have a large p-value for D-Calibration, which indicates that the distribution of observed proportions of events is statistically similar to the proportion of predicted proportions. Haider *et al.* provides a method to incorporate censored individuals into the D-calibration calculation by appropriately “spreading” each censored individual among the relevant time intervals [16].

B.3 L1-Margin Loss

L1-loss is another metric to compare survival prediction models by computing the difference between the observed event time and the predicted median survival time for uncensored instances. To incorporate censored instances into L1-loss calculation, we used the L1-Margin variant of this loss as described in [16]. For an individual censored at time c_i, L1-Margin loss sets the event time for this patient as

$$BG(c_i) \quad = \quad c_i \ + \ \frac{\int_{c_i}^{\infty} S_{KM}(t)\ dt}{S_{KM}(c_i)} \tag{5}$$

which is a “Best-Guess” value based on the censored time c_i , which uses the Kaplan-Meier curve estimated from the training dataset, $S_{KM}(t)$. (Note this corresponds to the expected value, given the survival time is at least c_i – see [16, Theorem B.1] However, this “Best-Guess” survival time can be more meaningful for some instances than others, based on the censored time c_i. For example, we know effectively nothing about a patient x_e censored at time $c_e = 0$ – meaning we should have very little confidence that $BG(0)$ matches x_e’s true survival time. By contrast, imagine no patients lived more than 1000 days in our dataset. If patient x_k was censored at time $c_k = 995$ (*i.e.*, close to this largest known survival time), we are fairly confident that this $BG(c_k)$ is close to the observed event time. Therefore, Haider *et al.*[16] set a confidence weight $\alpha_k = 1 - S_{KM}(c_k)$ for each “Best-Guess” estimation, which yields lower confidence for early censoring data and higher confidence for late censoring data.

B.3.1 Marginal Concordance index

All censored individuals could be included into the C-index calculation by using the aforementioned "Best-Guess"-value-based de-censoring approach, Equation 5. We define marginal C-index (mC-index) as the C-index variant that includes all pairs of censored and uncensored (after de-censoring) individuals into its calculation. Moreover, each pair of individuals in the mC-index calculation is assigned a confidence weight of 1 for pairs that were comparable in the original definition, $1 - S_{KM}(c_i)$ for original incomparable pairs with one censored individual, i, and $(1 - S_{KM}(c_i)) \cdot (1 - S_{KM}(c_j))$ for original incomparable pairs with two censored individuals, i and j. Note that all results reported in the main paper and the Appendix use the standard C-index to be comparable with many other papers; the mC-index results are provided only in the supplementary material.

C Additional Results

Table C.1 (resp., Table C.2, Table C.3) contains the quantitative performance results with respect to Figure 3 (resp., Figure 4, Figure 5) in the main text.

For patient mortality as the event of interest in dataset D2 – *i.e.*, D2[d] – all models were calibrated and RSF had marginally better C-index than the GBCM-KP model while having a worse L1-Margin loss; see Table C.4. Although MTLR exhibits the lowest L1-Margin loss among all the models, its C-index performance is not as good as RSF's and GBCM-KP's. We obtained substantially better results when we considered death as the event of interest instead of hospital discharge in dataset D2 for all models; this is probably due to the relatively high censoring rate for patient mortality as shown in Table 1 in the main text.

Table C.1. **Survival prediction performance on dataset D1[h] (N = 75063) for using age and sex as covariates, to predict time until hospital discharges.**

Model	C-index ± std	D-Calibration	L1-Margin Loss ± std
RSF	0.798 ± 0.011	**0.991**	165.553 ± 6.162
Cox-KP	0.822 ± 0.005	**0.999**	181.533 ± 9.533
MTLR	0.823 ± 0.005	**0.999**	**101.341 ± 10.622**
GBCM-KP	**0.827 ± 0.004**	**0.999**	193.437 ± 9.320

Table C.2. **Survival prediction performance on dataset D2[h] (N = 1718) for hospital discharge as event of interest with age, sex, and with/without chronic disease (binary indicator), longitude, and latitude as covariates.**

Model	Covariants	C-index ± std	D-Calibration	L1-Margin Loss ± std
RSF	age and sex	0.748 ± 0.030	**0.111**	13.149 ± 2.193
	5 covariants	**0.896 ± 0.038**	0	19.705 ± 4.312
Cox-KP	age and sex	0.682 ± 0.058	**0.998**	5.239 ± 0.872
	5 covariants	0.694 ± 0.047	**0.998**	5.846 ± 1.414
MTLR	age and sex	0.676 ± 0.058	**0.999**	5.481 ± 1.160
	5 covariants	0.691 ± 0.039	**0.999**	5.745 ± 1.505
GBCM-KP	age and sex	0.721 ± 0.081	**0.998**	4.767 ± 1.035
	5 covariants	**0.744 ± 0.052**	**0.999**	**4.120 ± 1.093**

Table C.5 and Table C.6 present the average performance of 5CV results on dataset D2 for hospital discharge and death as the event, respectively, after transforming the latitude and longitude variable to the Cartesian coordinates (see Eq 1 in the main text). Compared to the non-transformed results (Table C.2 vs. Table C.5, and Table C.4 vs. Table C.6), the models based on transformed features yield similar results.

For patient mortality as an event of interest in dataset D3 – D3[d] – including PD and GDP as meta information only marginally improved the results of all survival models as shown in Table C.7. The results of ISD models for mortality (Table C.7) are not impacted as significantly as the results for hospital discharge (Table C.3) due to relatively high censoring.

Table C.3. **Survival prediction performance on dataset D3[h] (N = 1422) for hospital discharge as event of interest with age, sex, chronic disease (binary indicator), longitude, latitude, and with/without population density (PD) and gross domestic product (GDP) as covariates.**

Model	wrt PD and GDP	C-index ± std	D-Calibration	L1-Margin Loss ± std
RSF	without	0.771 ± 0.023	**0.675**	5.652 ± 0.257
	with	**0.912 ± 0.045**	**0.108**	19.350 ± 4.123
Cox-KP	without	0.680 ± 0.046	**0.997**	6.003 ± 1.673
	with	0.707 ± 0.052	**0.999**	4.971 ± 1.214
MTLR	without	0.693 ± 0.052	**0.999**	6.353 ± 1.371
	with	0.739 ± 0.045	**0.999**	6.301 ± 2.139
GBCM-KP	without	0.787 ± 0.032	**0.998**	4.115 ± 1.223
	with	0.778 ± 0.050	**0.992**	**3.824 ± 1.179**

Table C.4. **Survival prediction performance on dataset D2[d] (N = 1718) for death as event of interest with age, sex, and with/without chronic disease (binary indicator), longitude, and latitude as covariates.**

Model	Covariants	C-index ± std	D-Calibration	L1-Margin Loss ± std
RSF	age and sex	0.804 ± 0.069	**0.999**	12.348 ± 15.923
	5 covariants	**0.967 ± 0.008**	**0.999**	12.021 ± 17.418
Cox-KP	age and sex	0.902 ± 0.033	**0.999**	11.049 ± 14.108
	5 covariants	0.903 ± 0.055	**0.999**	11.037 ± 14.304
MTLR	age and sex	0.910 ± 0.038	**0.999**	8.552 ± 5.232
	5 covariants	0.935 ± 0.022	**0.999**	**8.122 ± 4.888**
GBCM-KP	age and sex	0.917 ± 0.035	**0.999**	12.425 ± 16.243
	5 covariants	**0.965 ± 0.005**	**0.999**	10.756 ± 15.973

Table C.5. **Survival prediction performance on dataset D2[h] (N = 1718) for hospital discharge as event of interest with age, sex, chronic disease (binary indicator), and Cartesian coordinates transformation for location information (Eq 1) as covariates.**

Model	C-index ± std	D-Calibration	L1-Margin Loss ± std
RSF	**0.863 ± 0.062**	0	19.334 ± 4.208
Cox-KP	0.674 ± 0.062	**0.998**	4.981 ± 1.072
MTLR	0.666 ± 0.054	**0.999**	6.216 ± 1.859
GBCM-KP	0.732 ± 0.091	**0.998**	**4.458 ± 0.847**

Table C.6. **Survival prediction performance on dataset D2[d] (N = 1718) for death as event of interest with age, sex, chronic disease (binary indicator), and Cartesian coordinates transformation for location information (Eq 1) as covariates.**

Model	C-index ± std	D-Calibration	L1-Margin Loss ± std
RSF	**0.964 ± 0.009**	**0.999**	11.91 ± 17.037
Cox-KP	0.916 ± 0.045	**0.991**	**9.068 ± 11.622**
MTLR	0.939 ± 0.023	**0.998**	18.885 ± 13.429
GBCM-KP	**0.965 ± 0.007**	**0.999**	11.364 ± 17.307

Table C.7. **Survival prediction performance on dataset D3[d] (N = 1422) for death as event of interest with age, sex, chronic disease (binary indicator), longitude, latitude, and and with/without population density (PD) and gross domestic product (GDP) as covariates.**

Model	wrt PD and GDP	C-index ± std	D-Calibration	L1-Margin Loss ± std
RSF	without	0.960 ± 0.012	**0.990**	14.174 ± 7.176
	with	0.961 ± 0.011	**0.999**	11.628 ± 15.787
Cox-KP	without	0.940 ± 0.007	**0.999**	10.359 ± 13.689
	with	0.945 ± 0.004	**0.990**	**9.380 ± 11.658**
MTLR	without	0.941 ± 0.009	**0.999**	11.953 ± 13.685
	with	0.944 ± 0.009	**0.999**	12.572 ± 12.295
GBCM-KP	without	**0.962 ± 0.010**	**0.999**	9.906 ± 13.608
	with	**0.962 ± 0.009**	**0.999**	10.089 ± 14.031

Table C.8. **All ISD Model Comparisons**

	**Model**	**C-Index ± std**	**D-Calibration**	**L1-Margin Loss ± std**	**mC-index ± std**
D1[h]	Endpoint: Hospital Discharge	Covariate: Age and Sex			
	RSF	0.798 ± 0.011	**0.991**	165.553 ± 6.162	0.754 ± 0.013
	CoxKP	0.822 ± 0.005	**0.999**	181.533 ± 9.533	0.787 ± 0.010

	MTLR	0.823 ± 0.005	**0.999**	**101.341 ± 10.622**	0.790 ± 0.012
	GBCM-KP	**0.827 ± 0.004**	**0.999**	193.437 ± 9.320	0.785 ± 0.012
	AFT	0.821 ± 0.009	**0.759**	**94.619 ± 3.426**	0.790 ± 0.011
	PC-Hazard	0.794 ± 0.011	0	107.108 ± 4.000	0.792 ± 0.011
	Cox-Time	0.793 ± 0.011	**0.999**	84.321 ± 3.317	0.791 ± 0.011
	DeepHit	0.796 ± 0.011	0	91.290 ± 4.041	0.793 ± 0.011
D2[h]	Endpoint: Hospital Discharge	Covariate: Age, Sex, Location[Long+Lat] and Chronic disease			
	RSF	**0.896 ± 0.038**	0.000	19.705 ± 4.312	**0.694 ± 0.053**
	CoxKP	0.694 ± 0.047	**0.998**	5.846 ± 1.414	0.583 ± 0.030
	MTLR	0.691 ± 0.039	**0.999**	5.745 ± 1.505	0.595 ± 0.028
	GBCM-KP	**0.744 ± 0.052**	**0.999**	**4.120 ± 1.093**	**0.630 ± 0.038**
	AFT	0.693 ± 0.034	**0.999**	5.170 ± 1.149	0.580 ± 0.031
	PC-Hazard	0.683 ± 0.061	0	11.145 ± 0.324	0.620 ± 0.041
	Cox-Time	0.669 ± 0.061	**0.999**	4.881 ± 0.399	0.604 ± 0.041
	DeepHit	0.634 ± 0.090	**0.958**	4.789 ± 0.343	0.585 ± 0.067
D2[h]	Endpoint: Hospital Discharge	Covariate: Age, Sex, Tranformed Location and Chronic disease			
	RSF	**0.863 ± 0.062**	0.000	19.334 ± 4.208	**0.689 ± 0.046**
	CoxKP	0.674 ± 0.062	**0.998**	4.981 ± 1.072	0.568 ± 0.029
	MTLR	0.666 ± 0.054	**0.999**	6.216 ± 1.859	0.583 ± 0.037
	GBCM-KP	**0.732 ± 0.091**	**0.998**	**4.458 ± 0.847**	**0.624 ± 0.061**
	AFT	0.670 ± 0.058	**0.998**	5.407 ± 0.545	0.584 ± 0.031
	PC-Hazard	0.681 ± 0.071	0	11.181 ± 0.332	0.616 ± 0.049
	Cox-Time	0.659 ± 0.057	**0.999**	4.964 ± 0.301	0.598 ± 0.036
	DeepHit	0.659 ± 0.079	**0.988**	4.717 ± 0.311	0.603 ± 0.055
D2[h]	Endpoint: Hospital Discharge	Covariate: Age, Sex			
	RSF	**0.748 ± 0.030**	**0.111**	13.149 ± 2.193	0.623 ± 0.037
	CoxKP	0.682 ± 0.058	**0.998**	5.239 ± 0.872	0.596 ± 0.030
	MTLR	0.676 ± 0.058	**0.999**	5.481 ± 1.160	0.589 ± 0.037
	GBCM-KP	0.721 ± 0.081	**0.998**	**4.767 ± 1.035**	**0.634 ± 0.052**
	AFT	0.684 ± 0.055	**0.999**	4.987 ± 0.933	0.597 ± 0.030
	PC-Hazard	0.683 ± 0.061	0	11.145 ± 0.324	0.620 ± 0.041
	Cox-Time	0.655 ± 0.061	**0.999**	4.929 ± 0.164	0.592 ± 0.039
	DeepHit	0.645 ± 0.063	**0.966**	4.729 ± 0.242	0.592 ± 0.048
D3[h]	Endpoint: Hospital Discharge	Covariate: Age, Sex, Location[Long+Lat] and Chronic disease			
	RSF	0.771 ± 0.023	**0.675**	5.652 ± 0.257	**0.713 ± 0.047**
	CoxKP	0.680 ± 0.046	**0.997**	6.003 ± 1.673	0.566 ± 0.033
	MTLR	0.693 ± 0.052	**0.999**	6.353 ± 1.371	0.570 ± 0.041
	GBCM-KP	**0.787 ± 0.032**	**0.998**	**4.115 ± 1.223**	0.654 ± 0.012
	AFT	0.699 ± 0.043	**0.996**	5.260 ± 1.257	0.579 ± 0.032
	PC-Hazard	0.704 ± 0.034	0	11.254 ± 0.368	0.629 ± 0.033
	Cox-Time	0.704 ± 0.038	**0.999**	4.712 ± 0.487	0.618 ± 0.031
	DeepHit	0.709 ± 0.050	**0.996**	4.607 ± 0.361	0.631 ± 0.037
D3[h]	Endpoint: Hospital Discharge	Covariate: Age, Sex, Location[Long+Lat] and Chronic disease and GDP			
	RSF	**0.912 ± 0.045**	**0.108**	19.350 ± 4.123	**0.739 ± 0.047**
	CoxKP	0.707 ± 0.052	**0.999**	4.971 ± 1.214	0.604 ± 0.077
	MTLR	0.739 ± 0.045	**0.999**	6.301 ± 2.139	0.626 ± 0.029
	GBCM-KP	0.778 ± 0.050	**0.992**	**3.824 ± 1.179**	0.633 ± 0.039
	AFT	0.755 ± 0.085	**0.988**	6.713 ± 1.660	0.615 ± 0.077
	PC-Hazard	0.708 ± 0.035	0	11.290 ± 0.309	0.628 ± 0.042
	Cox-Time	0.704 ± 0.034	**0.999**	4.671 ± 0.438	0.619 ± 0.033
	DeepHit	0.688 ± 0.047	**0.989**	4.641 ± 0.411	0.615 ± 0.035
D2[d]	Endpoint: death	Covariate: Age, Sex, Location[Long+Lat] and Chronic disease			

	RSF	0.965 ± 0.008	**0.999**	12.021 ± 17.418	0.872 ± 0.033
	CoxKP	0.902 ± 0.055	**0.999**	11.037 ± 14.304	0.839 ± 0.043
	MTLR	0.935 ± 0.022	**0.999**	**8.122 ± 4.888**	0.850 ± 0.028
	GBCM-KP	**0.967 ± 0.005**	**0.999**	10.756 ± 15.973	**0.899 ± 0.011**
	AFT	0.916 ± 0.049	**0.999**	12.023 ± 13.748	0.850 ± 0.041
	PC-Hazard	0.846 ± 0.026	0	17.647 ± 3.999	0.855 ± 0.035
	Cox-Time	0.831 ± 0.030	**0.999**	11.134 ± 4.112	0.839 ± 0.022
	DeepHit	0.846 ± 0.028	**0.995**	10.648 ± 4.715	0.854 ± 0.037
D2[d]	Endpoint: death	Covariate: Age, Sex, Tranformed Location and Chronic disease			
	RSF	0.964 ± 0.009	**0.999**	11.910 ± 17.037	0.865 ± 0.027
	CoxKP	0.916 ± 0.045	**0.991**	**9.068 ± 11.622**	0.836 ± 0.056
	MTLR	0.939 ± 0.023	**0.998**	18.885 ± 13.429	0.868 ± 0.014
	GBCM-KP	**0.965 ± 0.007**	**0.999**	11.364 ± 17.307	**0.894 ± 0.019**
	AFT	0.921 ± 0.036	**0.999**	11.452 ± 13.345	0.827 ± 0.069
	PC-Hazard	0.846 ± 0.026	0	17.647 ± 3.999	0.855 ± 0.035
	Cox-Time	0.841 ± 0.017	**0.999**	10.461 ± 4.411	0.851 ± 0.021
	DeepHit	0.846 ± 0.028	**0.995**	10.648 ± 4.715	0.854 ± 0.037
D2[d]	Endpoint: death	Covariate: Age, Sex			
	RSF	0.804 ± 0.069	**0.999**	12.348 ± 15.923	0.669 ± 0.123
	CoxKP	0.903 ± 0.025	**0.999**	11.049 ± 14.108	0.843 ± 0.020
	MTLR	0.910 ± 0.022	**0.999**	**8.552 ± 5.232**	0.837 ± 0.035
	GBCM-KP	**0.917 ± 0.028**	**0.999**	12.425 ± 16.243	**0.854 ± 0.034**
	AFT	0.908 ± 0.022	**0.999**	11.994 ± 12.719	0.843 ± 0.023
	PC-Hazard	0.846 ± 0.026	0	17.647 ± 3.999	**0.855 ± 0.035**
	Cox-Time	0.844 ± 0.023	**0.999**	10.626 ± 4.521	0.853 ± 0.031
	DeepHit	0.836 ± 0.028	**0.993**	11.203 ± 4.395	0.842 ± 0.042
D3[d]	Endpoint: death	Covariate: Age, Sex, Location[Long+Lat] and Chronic disease			
	RSF	0.960 ± 0.012	**0.990**	14.174 ± 7.176	0.860 ± 0.020
	CoxKP	0.940 ± 0.007	**0.999**	10.359 ± 13.689	0.862 ± 0.012
	MTLR	0.941 ± 0.009	**0.999**	11.953 ± 13.685	0.859 ± 0.012
	GBCM-KP	**0.962 ± 0.010**	**0.999**	**9.906 ± 13.608**	**0.883 ± 0.016**
	AFT	0.939 ± 0.006	**0.999**	11.380 ± 12.557	0.858 ± 0.015
	PC-Hazard	0.840 ± 0.011	0	17.500 ± 3.391	0.836 ± 0.018
	Cox-Time	0.832 ± 0.017	**0.999**	10.658 ± 4.118	0.827 ± 0.023
	DeepHit	0.841 ± 0.018	**0.993**	10.631 ± 4.312	0.837 ± 0.028
D3[d]	Endpoint: death	Covariate: Age, Sex, Location[Long+Lat] and Chronic disease and GDP			
	RSF	0.961 ± 0.011	**0.999**	11.628 ± 15.787	0.863 ± 0.026
	CoxKP	0.945 ± 0.004	**0.990**	**9.380 ± 11.658**	0.872 ± 0.015
	MTLR	0.944 ± 0.009	**0.999**	12.572 ± 12.295	0.868 ± 0.013
	GBCM-KP	**0.962 ± 0.009**	**0.999**	10.089 ± 14.031	**0.883 ± 0.016**
	AFT	0.948 ± 0.005	**0.999**	12.079 ± 13.191	0.868 ± 0.014
	PC-Hazard	0.840 ± 0.011	0	17.500 ± 3.391	0.836 ± 0.018
	Cox-Time	0.837 ± 0.016	**0.999**	10.422 ± 4.394	0.833 ± 0.019
	DeepHit	0.821 ± 0.016	**0.995**	11.114 ± 3.997	0.819 ± 0.024

Table C.9. **AFT Distribution Comparison**

	**Model**	**C-Index ± std**	**D-Calibration**	**L1-Margin Loss ± std**	**mC-index ± std**
D1[h]	Endpoint: Hospital Discharge	Covariate: Age and Sex			
	Weibull	0.800 ± 0.008	**0.999**	130.562 ± 18.065	0.790 ± 0.012
	Exponential	**0.821 ± 0.009**	0.007	152.104 ± 5.425	0.788 ± 0.010
	Gaussian	0.820 ± 0.009	**0.987**	167.767 ± 8.148	0.790 ± 0.011

	Lognormal	**0.821 ± 0.009**	**0.759**	**94.619 ± 3.426**	0.790 ± 0.011
	Loglogistic	0.821 ± 0.009	**0.999**	111.173 ± 4.275	0.790 ± 0.011
D2[h]	Endpoint: Hospital Discharge	Covariate: Age, Sex, Location[Long+Lat] and Chronic disease			
	Weibull	0.669 ± 0.074	**0.992**	5.378 ± 1.188	0.565 ± 0.069
	Exponential	0.694 ± 0.042	0	18.178 ± 3.892	0.536 ± 0.063
	Gaussian	**0.697 ± 0.032**	**0.993**	**4.408 ± 1.133**	0.580 ± 0.035
	Lognormal	0.693 ± 0.034	**0.999**	5.170 ± 1.149	0.580 ± 0.031
	Loglogistic	0.686 ± 0.057	**0.999**	5.124 ± 1.164	0.575 ± 0.032
D2[h]	Endpoint: Hospital Discharge	Covariate: Age, Sex, Tranformed Location and Chronic disease			
	Weibull	0.648 ± 0.056	**0.995**	6.091 ± 0.732	0.565 ± 0.069
	Exponential	0.621 ± 0.058	0	16.331 ± 2.941	0.564 ± 0.028
	Gaussian	0.660 ± 0.056	**0.999**	**4.618 ± 0.687**	0.573 ± 0.026
	Lognormal	**0.670 ± 0.058**	**0.998**	5.407 ± 0.545	0.584 ± 0.031
	Loglogistic	0.662 ± 0.057	**0.993**	5.324 ± 0.635	0.573 ± 0.030
D2[h]	Endpoint: Hospital Discharge	Covariate: Age, Sex			
	Weibull	0.666 ± 0.065	**0.131**	4.603 ± 0.969	0.587 ± 0.041
	Exponential	0.679 ± 0.060	0	16.371 ± 3.242	0.590 ± 0.040
	Gaussian	0.676 ± 0.054	**0.997**	**4.145 ± 1.028**	0.591 ± 0.030
	Lognormal	**0.684 ± 0.055**	**0.999**	4.987 ± 0.933	0.597 ± 0.030
	Loglogistic	0.680 ± 0.058	**0.998**	4.905 ± 1.020	0.593 ± 0.032
D3[h]	Endpoint: Hospital Discharge	Covariate: Age, Sex, Location[Long+Lat] and Chronic disease			
	Weibull	0.664 ± 0.059	**0.971**	5.702 ± 1.26	0.554 ± 0.045
	Exponential	0.659 ± 0.047	0.004	18.320 ± 4.365	0.532 ± 0.035
	Gaussian	**0.701 ± 0.042**	**0.997**	**4.479 ± 1.348**	0.577 ± 0.031
	Lognormal	0.699 ± 0.043	**0.996**	5.260 ± 1.257	0.579 ± 0.032
	Loglogistic	0.689 ± 0.049	**0.993**	5.225 ± 1.273	0.569 ± 0.037
D3[h]	Endpoint: Hospital Discharge	Covariate: Age, Sex, Location[Long+Lat] and Chronic disease and GDP			
	Weibull	0.746 ± 0.085	**0.944**	7.436 ± 1.575	0.607 ± 0.076
	Exponential	0.707 ± 0.081	**0.167**	18.624 ± 4.474	0.552 ± 0.079
	Gaussian	0.746 ± 0.083	**0.993**	**4.833 ± 1.425**	0.603 ± 0.073
	Lognormal	**0.755 ± 0.085**	**0.988**	6.713 ± 1.660	0.615 ± 0.077
	Loglogistic	0.748 ± 0.087	**0.988**	7.307 ± 1.772	0.609 ± 0.077
D2[d]	Endpoint: death	Covariate: Age, Sex, Location[Long+Lat] and Chronic disease			
	Weibull	0.900 ± 0.056	**0.999**	**11.965 ± 12.968**	0.840 ± 0.039
	Exponential	**0.916 ± 0.051**	**0.999**	12.279 ± 16.247	0.734 ± 0.108
	Gaussian	0.913 ± 0.046	**0.999**	12.220 ± 9.830	0.850 ± 0.037
	Lognormal	**0.916 ± 0.049**	**0.999**	12.023 ± 13.748	0.850 ± 0.041
	Loglogistic	0.914 ± 0.050	**0.999**	12.222 ± 13.593	0.853 ± 0.035
D2[d]	Endpoint: death	Covariate: Age, Sex, Tranformed Location and Chronic disease			
	Weibull	0.897 ± 0.074	**0.999**	**11.246 ± 12.677**	0.816 ± 0.085
	Exponential	0.913 ± 0.052	**0.999**	**11.246 ± 12.677**	0.743 ± 0.133
	Gaussian	0.919 ± 0.044	**0.999**	11.510 ± 9.719	0.851 ± 0.033
	Lognormal	**0.921 ± 0.036**	**0.999**	11.452 ± 13.345	0.827 ± 0.069
	Loglogistic	0.902 ± 0.069	**0.999**	11.478 ± 12.953	0.819 ± 0.082
D2[d]	Endpoint: death	Covariate: Age, Sex			
	Weibull	0.907 ± 0.022	**0.999**	12.316 ± 12.931	0.845 ± 0.019
	Exponential	**0.908 ± 0.023**	**0.999**	12.02 ± 15.482	0.733 ± 0.097
	Gaussian	**0.908 ± 0.022**	**0.999**	13.027 ± 9.662	0.847 ± 0.020
	Lognormal	**0.908 ± 0.022**	**0.999**	**11.994 ± 12.719**	0.843 ± 0.023
	Loglogistic	**0.908 ± 0.022**	**0.999**	12.572 ± 12.829	0.846 ± 0.020
D3[d]	Endpoint: death	Covariate: Age, Sex, Location[Long+Lat] and Chronic disease			
	Weibull	0.937 ± 0.007	**0.999**	11.514 ± 12.392	0.856 ± 0.013

	Exponential	0.939 ± 0.006	**0.996**	**11.250 ± 13.444**	0.774 ± 0.095
	Gaussian	0.934 ± 0.005	**0.999**	12.264 ± 10.538	0.859 ± 0.012
	Lognormal	**0.940 ± 0.006**	**0.999**	11.380 ± 12.557	0.858 ± 0.015
	Loglogistic	0.937 ± 0.007	**0.999**	11.735 ± 12.543	0.857 ± 0.013
D3[d]	Endpoint: death	Covariate: Age, Sex, Location[Long+Lat] and Chronic disease and GDP			
	Weibull	0.943 ± 0.008	**0.999**	12.249 ± 13.419	0.860 ± 0.022
	Exponential	**0.948 ± 0.005**	**0.999**	**11.710 ± 14.545**	0.837 ± 0.025
	Gaussian	0.943 ± 0.008	**0.999**	12.799 ± 11.522	0.871 ± 0.016
	Lognormal	**0.948 ± 0.005**	**0.999**	12.079 ± 13.191	0.868 ± 0.014
	Loglogistic	0.947 ± 0.005	**0.999**	12.270 ± 13.175	0.868 ± 0.013

Table C.10. GBCM-Breslow vs. GBCM-KP Comparisons

	**Model**	**C-Index ± std**	**D-Calibration**	**L1-Margin Loss ± std**	**mC-index ± std**
D1[h]	Endpoint: Hospital Discharge	Covariate: Age and Sex			
	Kalbfleisch-Prentice	0.827 ± 0.004	0.999	**193.437 ± 9.320**	0.785 ± 0.012
	Breslow	0.827 ± 0.004	0.999	195.963 ± 9.438	0.785 ± 0.012
D2[h]	Endpoint: Hospital Discharge	Covariate: Age, Sex, Location[Long+Lat] and Chronic disease			
	Kalbfleisch-Prentice	0.744 ± 0.052	0.999	4.120 ± 1.093	0.630 ± 0.038
	Breslow	0.744 ± 0.052	0.999	**4.013 ± 1.012**	0.630 ± 0.038
D2[h]	Endpoint: Hospital Discharge	Covariate: Age, Sex, Tranformed Location and Chronic disease			
	Kalbfleisch-Prentice	0.732 ± 0.091	0.998	4.458 ± 0.847	0.624 ± 0.061
	Breslow	0.732 ± 0.091	0.998	**4.356 ± 0.850**	0.624 ± 0.061
D2[h]	Endpoint: Hospital Discharge	Covariate: Age, Sex			
	Kalbfleisch-Prentice	0.721 ± 0.081	0.998	4.767 ± 1.035	0.634 ± 0.052
	Breslow	0.721 ± 0.081	0.999	**4.584 ± 0.997**	0.634 ± 0.052
D3[h]	Endpoint: Hospital Discharge	Covariate: Age, Sex, Location[Long+Lat] and Chronic disease			
	Kalbfleisch-Prentice	0.787 ± 0.032	0.998	4.115 ± 1.223	0.654 ± 0.012
	Breslow	0.787 ± 0.032	0.999	**4.050 ± 1.108**	0.654 ± 0.012
D3[h]	Endpoint: Hospital Discharge	Covariate: Age, Sex, Location[Long+Lat] and Chronic disease and GDP			
	Kalbfleisch-Prentice	0.778 ± 0.050	0.992	**3.824 ± 1.179**	0.633 ± 0.039
	Breslow	0.778 ± 0.050	0.995	3.883 ± 1.157	0.633 ± 0.039
D2[d]	Endpoint: death	Covariate: Age, Sex, Location[Long+Lat] and Chronic disease			
	Kalbfleisch-Prentice	0.967 ± 0.005	0.999	**10.756 ± 15.973**	0.899 ± 0.011
	Breslow	0.967 ± 0.005	0.999	12.306 ± 15.973	0.899 ± 0.011
D2[d]	Endpoint: death	Covariate: Age, Sex, Tranformed Location and Chronic disease			
	Kalbfleisch-Prentice	0.965 ± 0.007	0.999	**11.364 ± 17.307**	0.894 ± 0.019
	Breslow	0.965 ± 0.007	0.999	12.721 ± 16.912	0.894 ± 0.019
D2[d]	Endpoint: death	Covariate: Age, Sex			
	Kalbfleisch-Prentice	0.917 ± 0.028	0.999	**12.425 ± 16.243**	0.854 ± 0.034
	Breslow	0.917 ± 0.028	0.999	14.814 ± 15.187	0.854 ± 0.034
D3[d]	Endpoint: death	Covariate: Age, Sex, Location[Long+Lat] and Chronic disease			
	Kalbfleisch-Prentice	0.962 ± 0.010	0.999	**9.906 ± 13.608**	0.883 ± 0.016
	Breslow	0.962 ± 0.010	0.999	11.467 ± 13.961	0.883 ± 0.016
D3[d]	Endpoint: death	Covariate: Age, Sex, Location[Long+Lat] and Chronic disease and GDP			
	Kalbfleisch-Prentice	0.962 ± 0.009	0.999	**10.089 ± 14.031**	0.883 ± 0.016
	Breslow	0.962 ± 0.009	0.999	11.457 ± 14.077	0.883 ± 0.016
